# Structural Insight into Terminal Galactose Recognition by Two Non-HBGA Binding GI.3 Noroviruses

**DOI:** 10.1128/jvi.00420-22

**Published:** 2022-06-06

**Authors:** Chenlong Wang, Huiling Kang, Ming Tan, Jingyuan Cong, Dan Su, Xuemei Li, Yutao Chen

**Affiliations:** a National Laboratory of Biomacromolecules, Institute of Biophysics, Chinese Academy of Sciences, Beijing, China; b School of Medicine, Tsinghua-Peking Center for Life Sciences, Tsinghua University, Beijing, China; c State Key Laboratory of Biotherapy, West China Hospital, Collaborative Innovation Center for Biotherapy, Sichuan University, Chengdu, China; d University of Chinese Academy of Sciences, Beijing, China; e Division of Infectious Diseases, Cincinnati Children’s Hospital Medical Center, Cincinnati, Ohio, USA; f Department of Pediatrics, University of Cincinnati College of Medicine, Cincinnati, Ohio, USA; University of Kentucky College of Medicine

**Keywords:** ELISA, HBGA, MST, P protein, capsid, complex, crystallography, norovirus

## Abstract

Human noroviruses (huNoVs) cause epidemic acute gastroenteritis using histo-blood group antigens (HBGAs) as host receptors or attachment factors to initiate an infection. While most huNoVs have been shown to bind HBGAs, some known clinical isolates, such as GI.3 DSV and VA115, do not recognize any HBGAs and thus the molecular mechanism behind their infections remains elusive. In this study, we provided both phenotypic and structural evidence to show that huNoV DSV and VA115 recognize a group of glycans with terminal galactoses as ligands. First, through glycan array we found that both DSV and VA115 protruding (P) domain proteins bound two oligosaccharides that share common terminal galactoses. Then, by determination of the crystal structures of DSV/VA115 P proteins in complex with Galα1-3Galβ1-4Glc and/or NA2 *N*-Glycan, respectively, we showed that the terminal galactose is the main saccharide recognized by the two viral proteins. Our data demonstrated that GI huNoVs can interact with non-HBGA glycans through their conserved galactose binding site, shedding light on the mechanism of huNoV adaptation through recognizing new glycan receptors to facilitate their widespread nature in human population. These findings are also of significance in strategy development for huNoV control and prevention, as well as development of antiviral drugs.

**IMPORTANCE** Human noroviruses (huNoVs) are the most important viral pathogens causing epidemic acute gastroenteritis worldwide. Previous studies indicated that histo-blood group antigens (HBGAs) are critical host-susceptibility factors affecting huNoV host susceptibility, host range, and probably prevalence. However, certain huNoVs, such as GI.3 DSV and VA115, do not recognize any HBGAs. This implies that other unknown host factors might exist and the molecular mechanism underlying their host receptor recognition or attachment remains elusive. In this study, we found that purified capsid protruding domain proteins from two GI.3 huNoVs specifically bind two glycans that contain a common terminal galactose. We solved the crystal structures of the complexes at atomic resolution and validated the vital amino acids involved in glycan recognition. Our findings elucidate the mechanism of GI.3 huNoV-non-HBGA glycan interaction, which explains why GI.3 virus strains could not bind human HBGAs, paving a way to the prevention and treatment of huNoV-associated diseases.

## INTRODUCTION

Noroviruses (NoVs), members of *Norovirus* genus in the family *Caliciviridae*, are the most important viral causative agents causing epidemic acute gastroenteritis worldwide (1–5). They are a group of nonenveloped RNA viruses with a diameter of 38–40 nm (6, 7) containing a single-stranded, positive-sense RNA genome of about 7.7 kb (8, 9). The NoV genomes consist of three open reading frames (ORFs), among which ORF1 encodes nonstructural proteins, ORF2 encodes viral protein 1 (VP1) that is the major capsid protein, while ORF3 encodes VP2 that is a minor capsid protein (8). NoV VP1 can be divided into two major domains: the N-terminal shell (S) domain and the C-terminal protruding (P) domain (10). The P domains form the outermost, dimeric protrusions of NoV virions (11, 12), which plays critical roles in receptor recognition and host immune responses.

Up to now ~305 NoVs strains have been discovered, which can be classified into 10 genogroups (GI-GX) and more than 40 genotypes (13). GI, GII, and GIV NoVs infect humans and thus are referred as human NoVs (huNoVs) (14). GII is the most widespread genogroup with GII.4 genotype circulating predominantly, causing majority of huNoV-associated clinical cases (15, 16), followed by GII.17, GII.3, and GII.6 genotypes. Meanwhile, GI.3 genotype is the most common GI huNoVs (17).

HuNoVs recognize human histo-blood group antigens (HBGAs) as host receptors or attachment factors that play an important role in huNoV host susceptibility. HBGAs are a group of fucose-containing glycans that are the major determinants of human blood types, including ABO, secretor (H), and Lewis blood types, and HBGAs also distribute abundantly on cell surface of mucosal epithelia (18). HuNoVs interact with HBGAs through the exterior P domains in complex patterns (19–23). The HBGA binding sites (HBSs) and the major binding saccharide (MaBS) are highly conserved within GI or GII huNoVs but are distinct between the two genogroups, where GI huNoVs interact with the β-galactose (β-Gal) of HBGAs as the MaBS and GII huNoVs recognize the α-fucose (α-Fuc) as MaBS (20, 22, 24, 25). Although the vast majority of huNoVs infect humans through recognizing HBGAs, a few huNoVs do not recognize HBGAs, such as GI.3 Desert Shield virus (DSV) (26, 27) and VA98115 (VA115) (28, 29), although some other GI.3 NoVs were resorted to bind HBGAs (30–32). The mechanism as to how these non-HBGA binding GI.3 NoVs bind the host cells to initiate an infection still remains unclear. Although VA115 was reported to bind gangliosides and sialylated carbohydrates (33, 34), the structural details were not clear yet.

To solve this puzzle, the P proteins of GI.3 DSV and VA115 were studied. Glycan array screening showed that two non-HBGA oligosaccharides interact with DSV and VA115 P proteins. Using X-ray crystallography, the structures of VA115 and DSV P proteins, as well as their complexes with the two non-HBGA oligosaccharides Galα1-3Galβ1-4Glc and NA2 *N*-Glycan, were solved at atomic resolution. The structural data showed that the two GI.3 huNoVs bound non-HBGA glycans using terminal Gal as the MaBS, explaining the mechanism why these huNoVs could not bind HBGAs. Our findings shed light on the complex recognition patterns of huNoV-glycan interactions, explaining why DSV and VA115 can infect humans without interacting with HBGAs.

## RESULTS

### Glycan array screenings of the two GI.3 P proteins.

**FIG 1:**
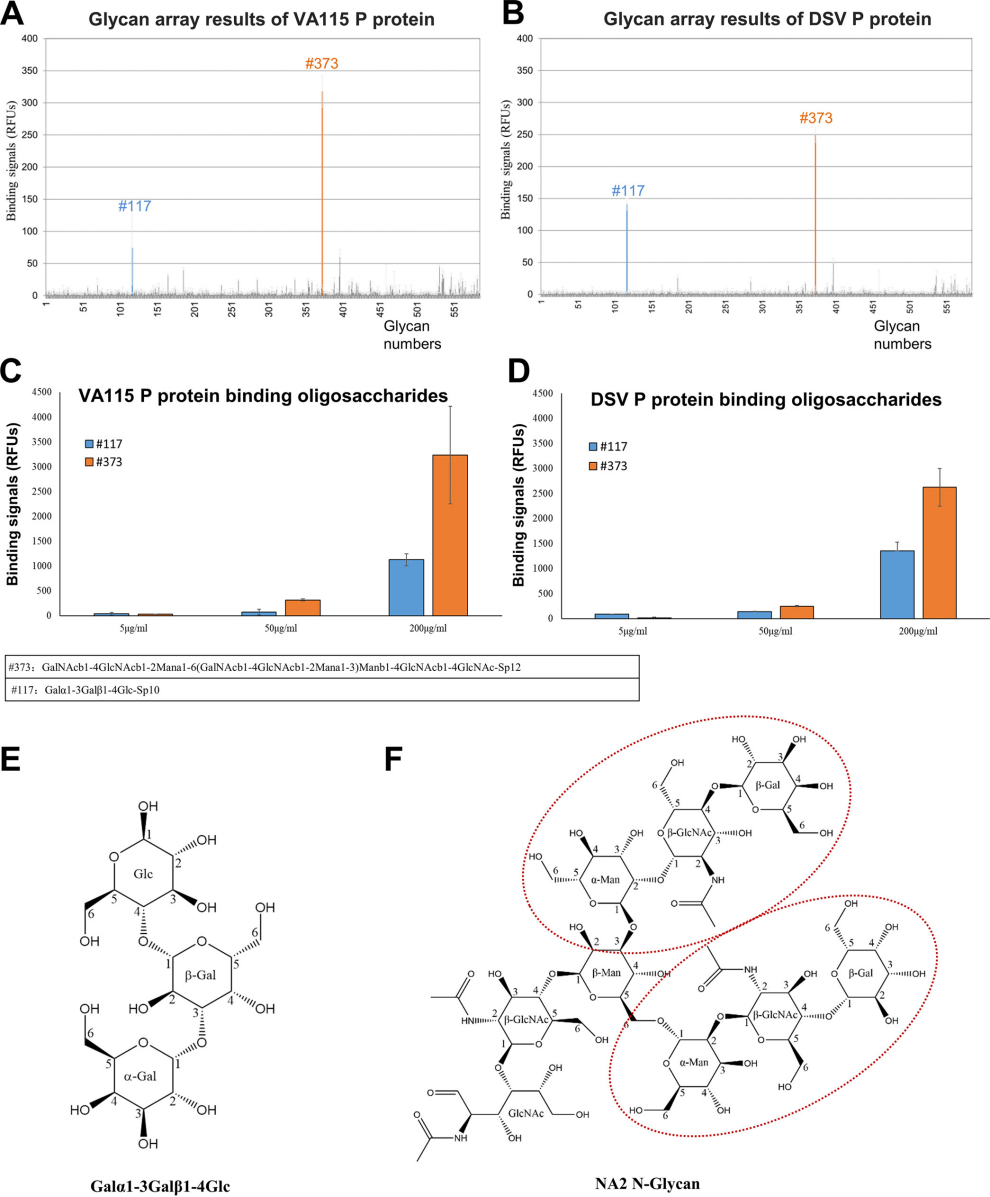
Glycan binding features of the GI.3 huNoV DSV and VA115 P proteins. (A and B) The results of a glycan array screening against a glycan library with 585 glycans aiming to identify glycan ligands to the VA115 (A) and DSV (B) P proteins. The binding signals to the glycans in average relative fluorescence units (RFUs) are shown in *y* axis, while the glycan numbers are shown in *x* axis. The two glycans that detected with a stronger binding signal were colored blue and orange, respectively. The detailed glycan array results are stored in the public database of NCFG with code 3872 and 3873 (http://www.functionalglycomics.org/glycomics/publicdata/selectedScreens.jsp). (C and D) The binding signals to the two oligosaccharides in average RFUs are shown by blue columns (no. 117) or orange columns (no. 373) in *y* axis with standard deviations in gray error bars, while the VA115 (C) and DSV (D) P proteins concentrations are shown in *x* axis. (E) Show the chemical structure of the detected Galα1-3Galβ1-4Glc (no. 117). (F) Chemical structure of NA2 *N*-Glycan, which is similar to the glycan GalNAcb1-4GlcNAcb1-2Mana1-6(GalNAcb1-4GlcNAcb1-2Mana1-3)Manb1-4GlcNAcb1-4GlcNAc-Sp12 (no. 117), and as a substitute to be soaked into protein crystal. It is an inverted Y-shape branched structure, with two ends composed of identical trisaccharides, which were indicated in dotted red circles.

Purified DSV/VA115 P proteins were screened against 585 mammalian cell-associated glycans, which included HBGA-related glycans to identify their possible glycan ligands or receptors. The results showed that two oligosaccharides (117 and 373) bound well to both DSV/VA115 P-GST fusion proteins with significantly higher binding signals compared with the remaining 583 oligosaccharides (Fig. 1A and B). Glycan binding assays showed that the binding strengths of the two oligosaccharides to the P proteins were concentration dependent, validating the binding specificity (Fig. 1C and 1D).

Glycan 117 is a trisaccharide (Galα1-3Galβ1-4Glc, CID in PubChem: 100962852), with a molecular weight of 504.4 Da, while the glycan 373 is oligosaccharide with sequences of GalNAcβ1-4GlcNAcβ1-2Mana1-6 (GalNAcβ1-4GlcNAcβ1-2Mana1-3)Manβ1-4GlcNAcβ1-4GlcNAc. Since the glycan 373 was unattainable, its analog NA2 *N*-Glycan (CID in PubChem: 71308690) with a molecular weight of 1,641.5 Da was used for complex formation in our crystallography study (see below). NA2 *N*-Glycan is composed of nine monosaccharides forming a Y-shaped branch structure, with two identical terminal trisaccharides. The two-dimensional structures of the two oligosaccharides are depicted in Fig. 1E and F.

### Crystal structures of native DSV and VA115 P proteins.

The crystal structure of the native DSV P protein was solved at 2.10 Å resolution, containing a homodimer in the crystallographic asymmetric unit (Fig. S1C in supplemental materials). All DSV P protein residues (228–531) were built in electronic density map. The crystal structure of native VA115 P protein was determined at 2.18 Å resolution, containing four VA115 P protein protomers or two homodimers in the crystallographic asymmetric unit (Fig. S1D). Most residues of VA115 P protein were modeled except residues S409–A415 (in B chain), due to uninterpretable local electron density maps caused by the local disorder of this highly flexible loop region.

The two P proteins exhibited the typical huNoV P dimer arch-like structure with the P1 subdomains situated at the bottom and the P2 subdomain situated at the top. The major structural differences compared with other known P proteins are at the surface exposed loops, including the previously identified A-, B-, P-, T-, and U-loops in the P2 subdomain and the S-loop in the P1 subdomain. These variable loops confer DSV and VA115 unique surface topology from other huNoVs (see Fig. 6).

### Structures of DSV and VA115 P proteins complexed with oligosaccharides.

**FIG 2:**
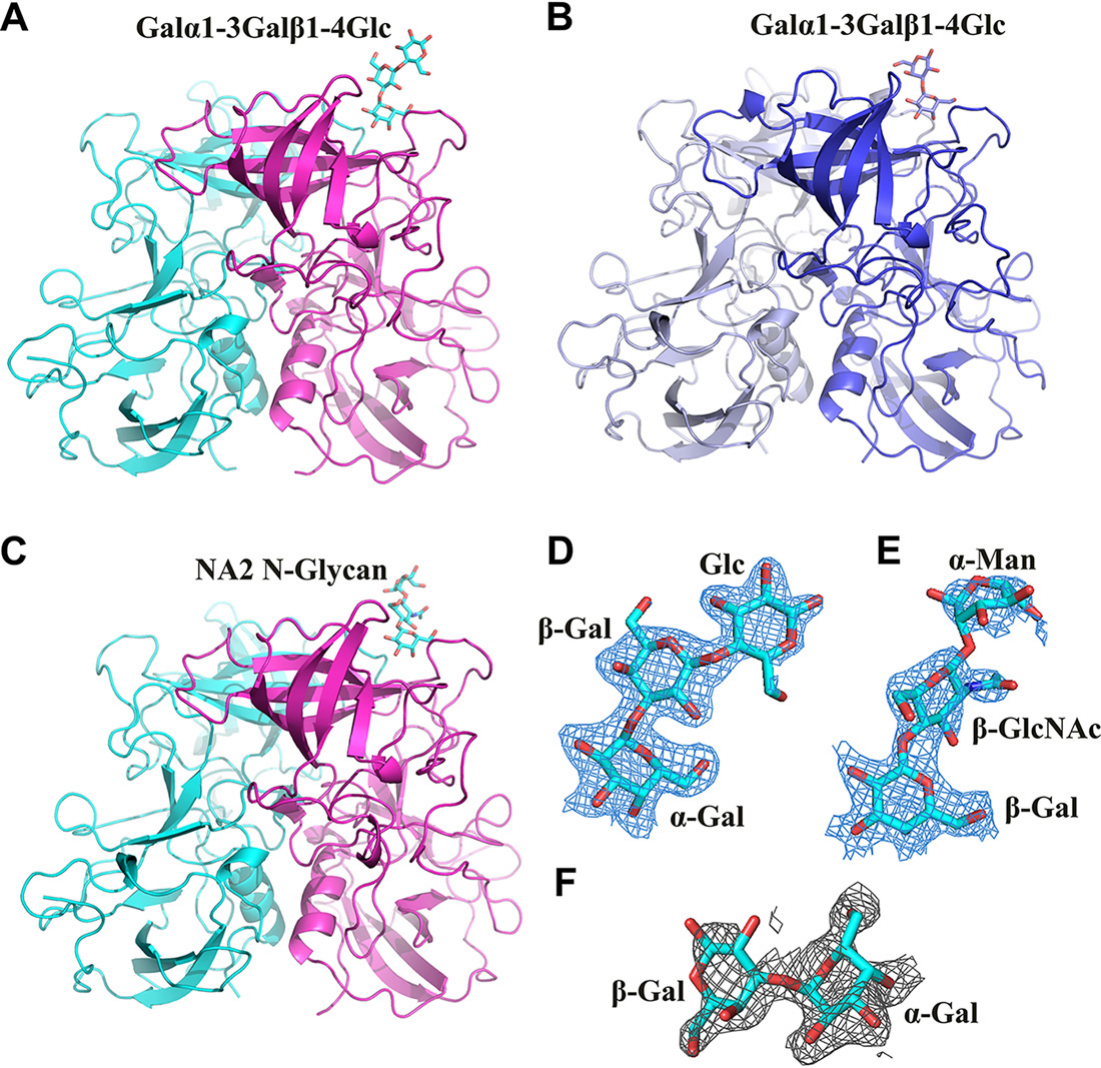
The crystal structures of GI.3 DSV and VA115 P protein in complex with oligosaccharides. (A and C) Cartoon representation of DSV P dimer with one protomer (light magenta) in complex with Galα1-3Galβ1-4Glc (A) and NA2 *N*-Glycan (C). (B) Cartoon representation of VA115 P dimer with one protomer (purple) in complex with Galα1-3Galβ1-4Glc. Galα1-3Galβ1-4Glc and NA2 *N*-Glycan are shown in stick representation. (D and E) (2mFo-DFc) omit electron density maps of the Galα1-3Galβ1-4Glc (D) and NA2 *N*-Glycan (E) in GI.3 DSV P protein complex structures, respectively. (F) (2mFo-DFc) omit electron density map of the Galα1-3Galβ1-4Glc in GI.3 VA115 P protein complex structure. The mesh map of Galα1-3Galβ1-4Glc or NA2 *N*-Glycan were contoured at 1σ, with a coverage at a 1.6-Å radius around glycans. Gal, galactose; GlcNAc, *N*-acetylglucosamine; Glc, glucose.

By crystal soaking, we determined complex structures of DSV P protein with Galα1-3Galβ1-4Glc and NA2 *N*-Glycan, respectively, and the complex structure of VA115 P protein with Galα1-3Galβ1-4Glc (Fig. 2A–C). The clear (2mFo-DFc) electron density omit maps showed the density of oligosaccharides at 1.0 σ contour level (Fig. 2D–F). Interestingly, only one oligosaccharide molecule bound in each asymmetric unit of the complex structures, since the remaining oligosaccharide binding pockets contain steric hindrance for oligosaccharides at crystal packing interface (Fig. S2). The density of the three-saccharide rings was clearly recognized and modeled in the 2.05 Å resolution structure of DSV P complexed with Galα1-3Galβ1-4Glc (Fig. 2D). Noteworthy, only the density of terminal trisaccharides (Galβ1-4 GlcNAcβ1-2αMan) could be modeled in the 2.15 Å structure of DSV P-NA2 *N*-Glycan complex (Fig. 2E), due to weak electron density of the remaining flexible parts. The same phenomenon was noted in the 2.20 Å structure of VA115 P protein-Galα1-3Galβ1-4Glc complex, where only the terminal disaccharides was determined (Fig. 2F). No complex was formed by soaking native VA115 P protein crystals with NA2 *N*-Glycan, probably due to crystal packing. In addition, since bile acids and monosialogangliosides were reported to bind huNoVs previously (33–37), we also tried cocrystalizing the two P proteins with bile acids and monosialogangliosides GM1 and GM3, but no complex was formed in the solved crystal structures.

### The glycan binding sites of DSV and VA115.

**FIG 3:**
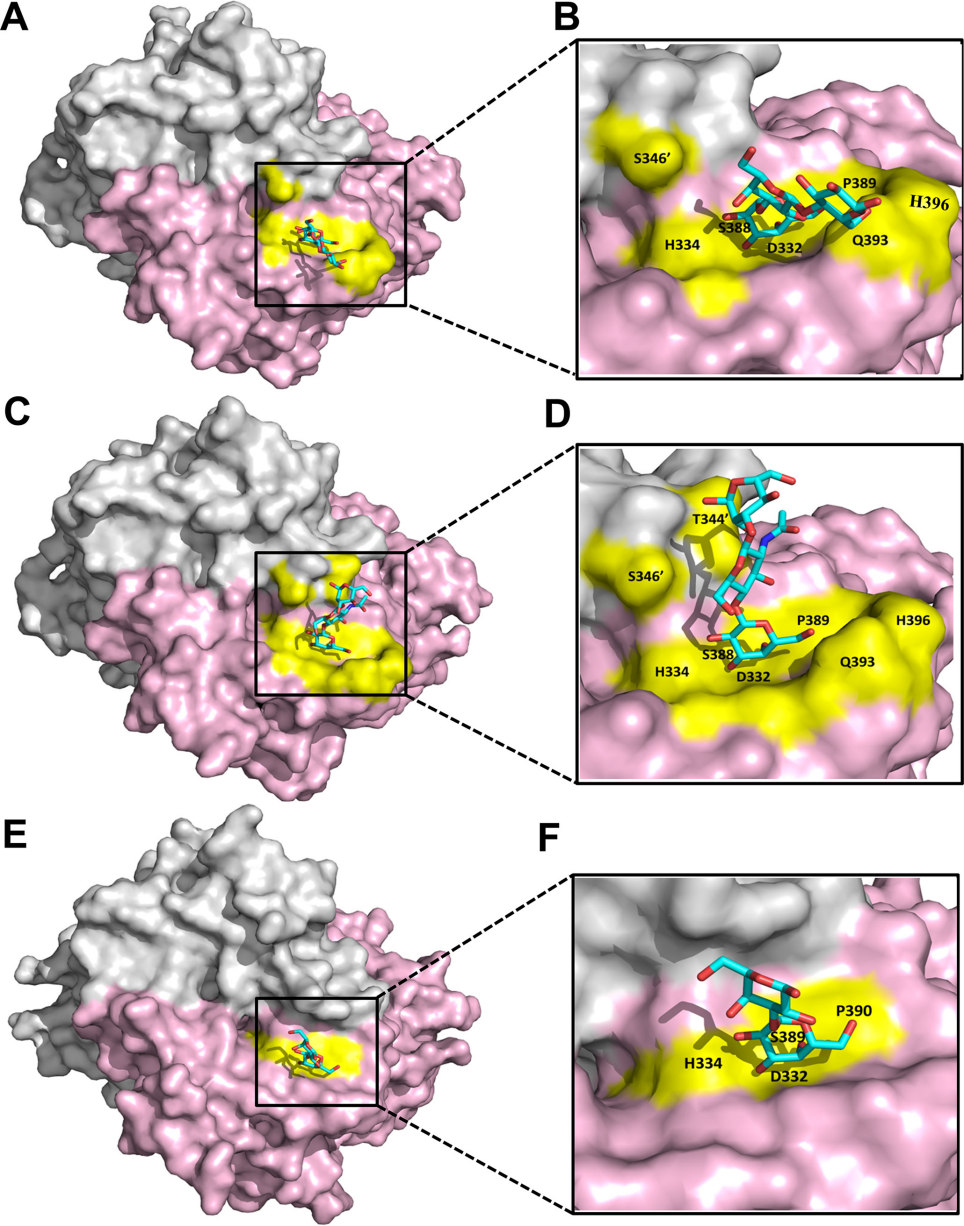
Localization and binding of glycans with GI.3 P dimers. (A to D) Top view of the DSV P dimer in complex with Galα1-3Galβ1-4Glc (A and B) and NA2 *N*-Glycan (C and D). (E and F) Top view of the VA115 P dimer in complex with Galα1-3Galβ1-4Glc. In A–F, the two P monomers (surface model) are in gray and light pink, respectively. The residues forming the binding interfaces are shown in yellow, while the bound Galα1-3Galβ1-4Glc and NA2 *N*-Glycan (stick model) are in cyan.

The positions of the glycan binding pockets were located at the outermost surface of the P protein near the interface between the two P monomers (Fig. 3), similar to the typical HBGA binding sites of other GI huNoVs (20–22, 38). In DSV P protein, four amino acids D332, H334, S388 and P389 form the “bottom” of the binding interface, while residues Q393 and H396 constitute a side “wall,” S346′ and T344′ from the other monomer form the opposite “wall” (Fig. 3A– D). The binding interface of VA115 P protein resembles that of DSV, in which its “bottom” is also composed of the four conserved residues D332, H334, S389, and P390 (Fig. 3E and F).

### The key role of galactose in interaction with DSV and VA115 P proteins.

**FIG 4:**
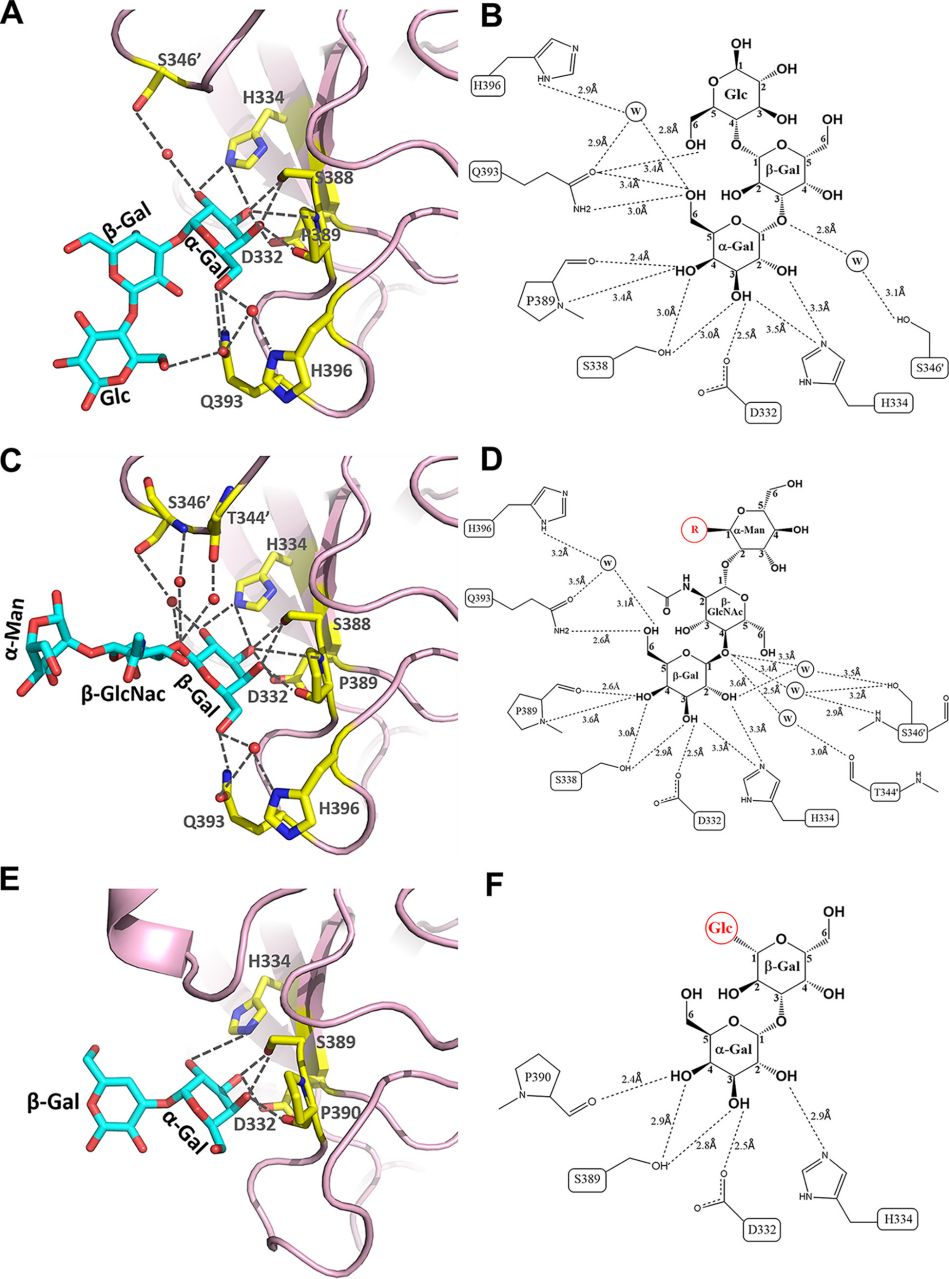
Detailed interaction networks between glycans and GI.3 P proteins. (A and C) The interaction networks between GI.3 DSV P protein and Galα1-3Galβ1-4Glc (A), or NA2 *N*-Glycan (C). (E) The interaction networks between GI.3 VA115 P protein and Galα1-3Galβ1-4Glc.The amino acids participating in those interactions are shown in stick representation in yellow, while the Galα1-3Galβ1-4Glc and NA2 *N*-Glycan with indications of the galactose (Gal), *N*-acetylglucosamine (GlcNAc), Mannose (Man) and glucose (Glc) shown as stick models in cyan. The hydrogen bonds are shown by gray dashed lines. (B and D) Schematic illustrations of the detail hydrogen bond (black dashed lines) network between amino acids of the DSV P protein binding interface and the individual saccharides of the Galα1-3Galβ1-4Glc (B) and NA2 N-Glycan (D), and involved water molecules are denoted by W. (F) Schematic illustrations of the detail hydrogen bond (black dashed lines) network between amino acids of the VA115 P protein binding interface and the individual saccharides of the Galα1-3Galβ1-4Glc.

**FIG 5:**
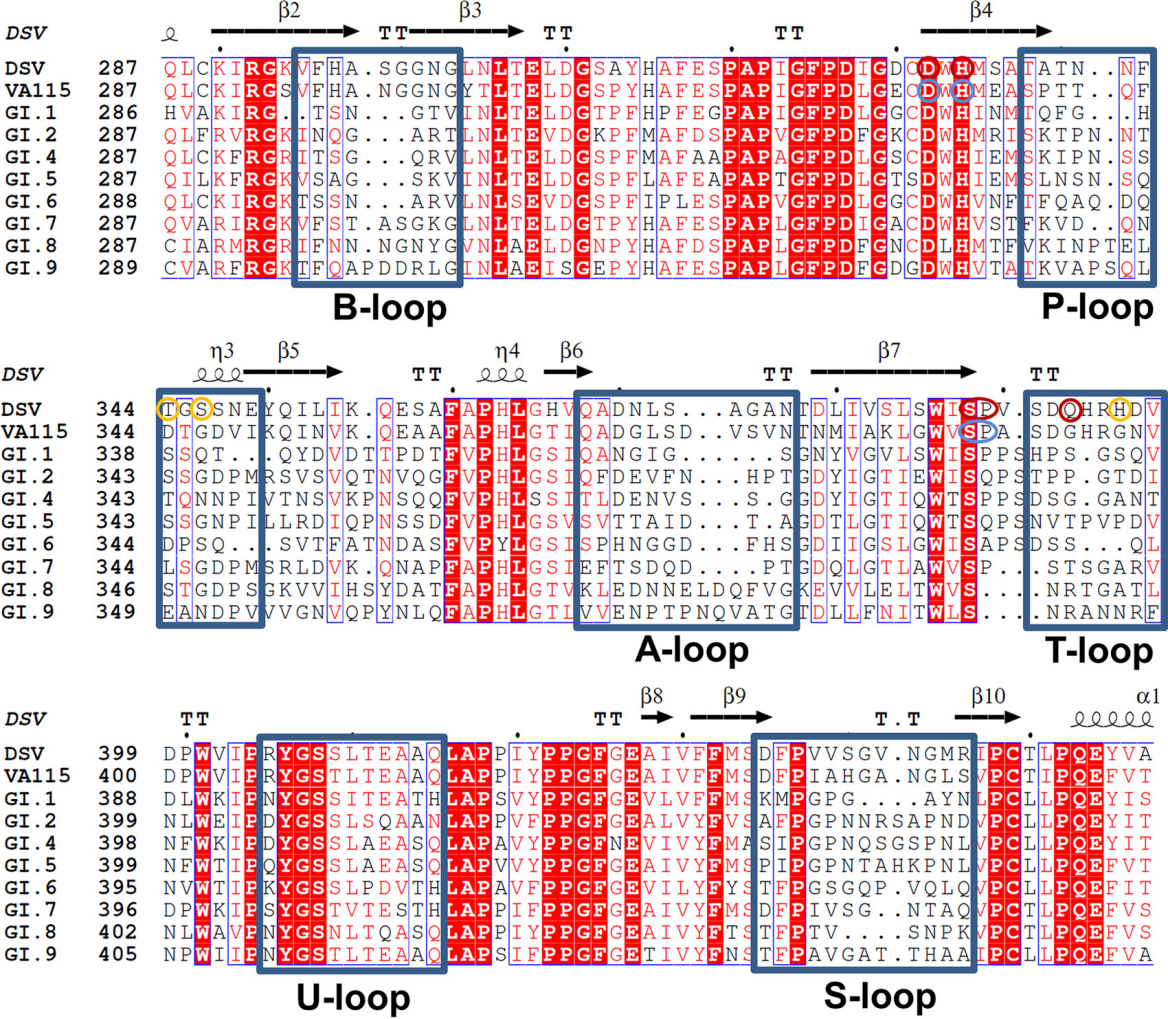
Sequence alignment analyses of GI.3 DSV and VA115 partial P domains among GI huNoVs. GI.1 NV, the prototype Norwalk virus (M87661); GI.2 FUV (BAC05516); GI.3 DSV, Desert Shield virus (AAA16285); GI.3 VA115, VA98115 (AAK84673); GI.4 Chiba, Chiba virus (BAB18267); GI.5 Musgro, Musgrove virus (AJ277614); GI.6 Wiscon, Wisconsin virus (AY502008); GI.7 TCH, TCH060 (AEQ77282); GI.8 Boxer, Boxer virus (AF538679); and GI.9 Lilla, Lilla Edet virus (AEY77023). The five surface loops (A-, B-, P-, S-, T-, and U- loops) are shown by blue rectangular frames. The residues involved in direct hydrogen bonds and water-mediated hydrogen bonds of the Galα1-3Galβ1-4Glc and NA2 *N*-Glycan binding interface of DSV P protein are highlighted by red and yellow circles, respectively. The residues involved in direct hydrogen bonds of the Galα1-3Galβ1-4Glc binding interface of VA115 P protein are highlighted by blue circles.

Both glycans share a terminal galactose (Gal) that dedicates the main interaction with the P proteins. In DSV the Gal interacts with all four conserved amino acids (D332, H334, S388, and P389), as well as a variable residue Q393, forming eight or seven direct hydrogen bonds (Fig. 4A–D). Additionally, the Gal indirectly interacts with H396 and S346′ through water molecules. Similarly, in VA115 the Gal forms five direct hydrogen bonds with four conserved amino acids, D332, H334, S389, and P390, as well as G394 at the position corresponding to Q393 of the DSV P protein that does not participate in direct interaction (Fig. 4E and 4F). Noteworthy, the four critical amino acids which play a key role in the observed interactions are highly conserved among GI huNoVs (Fig. 5).

### Validation of the glycan binding site by mutagenesis.

To verify the roles of the critical residues constituting the glycan binding sites, residues D332, H334, S388, P389, and Q393 of DSV P protein, as well as residues D332, H334, S389, and P390 of VA115 P protein were mutated to an alanine (A) individually. The interaction between native/mutant P proteins and the Galα1-3Galβ1-4Glc-HSA were measured by Micro-Scale Thermophoresis (MST) method. The results (Fig. S3) showed that most of the mutant P proteins lost their binding ability to the trisaccharide, confirming the indispensable roles of these amino acids in constituting the functional glycan binding sites of the two huNoVs. P390A of VA115 was the only mutation that did not change the glycan binding ability of the mutant P protein, presumably because this residue interacts with the trisaccharide through its main chain carbonyl group (Fig. S3A–E). Similarly, mutations D332A and S388A of DSV resulted in loss of the glycan binding ability, while mutations H334A and Q393A of DSV reduced the binding affinity. Mutation P389A of DSV also retains nearly full glycan binding ability, since this residue interacts with the trisaccharide through the main chain carbonyl group (Fig. S3F–K).

## DISCUSSION

Earlier studies indicated that huNoVs recognize HBGAs as attachment factors, in which huNoVs bind different HBGAs in a strain-specific manner (39, 40). The crystal structures of the HBGA binding interfaces of four GI NoVs, the GI.1 NV, the GI.2 FUV, the GI.7 TCH, and the GI.8 BV, have been previously determined (20–22, 38). In spite of great differences on the surface topology due to the sequence variations of the P domains, GI NoVs share similar overall structural features of the HBGA binding interfaces, where the amino acids involved in the glycan binding are conserved (Fig. 5). Nevertheless, GI.3 DSV and VA115 do not interact with any tested HBGA types in previous studies (28, 30), raising two key questions: (1) how did these two GI.3 huNoVs lose their HBGA binding ability, and (2) what might be the host factor(s) that facilitate infection of these GI.3 huNoVs.

The new structural data presented in this study have answered the questions at least partially. Through structural superimposition of the DSV P domain and HBGA oligosaccharides with other known structures (Fig. S4), we found that the α-acetylgalactosamine (GalNAc), the A epitope, and α-Gal of the B epitope are too close to the amino acids on the T-loop, resulting in an obvious chemical steric hindrance (Fig. S4A and B). Similar clash also occurs between the α-1,2 fucose of the H-type trisaccharide and the S346′ amino acid residue on the P-loop (Fig. S4C). Likewise, the representative α-1,3/4 fucose of Lewis antigen forms a clash with Y439′ on the S-loop (Fig. S4D–G).

**FIG 6:**
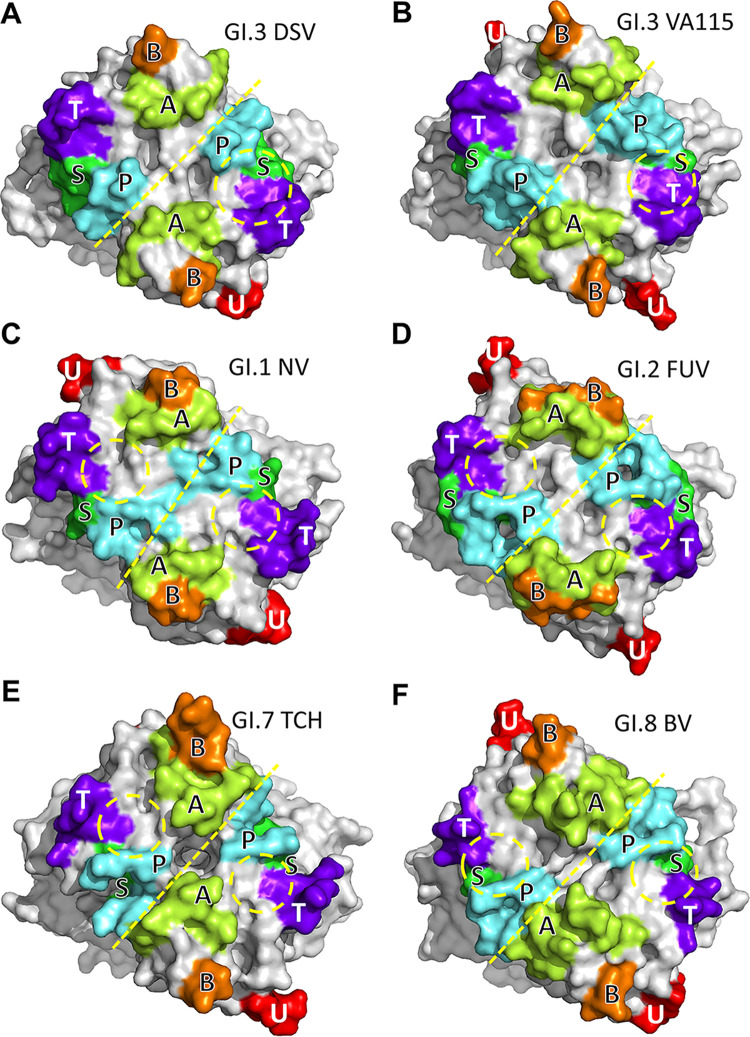
Comparisons of the surface structures of the six loops among the P dimers (surface models) of Desert Shield virus (GI.3 DSV; A), VA98115 (GI.3 VA115; B), Norwalk virus (GI.1 NV; C), FUV258 (GI.2 FUV; D), TCH060 (GI.7 TCH; E), and Boxer virus (GI.8 BV; F). The A-, B-, P-, S-, T-, and U-loop regions are indicated by different colors. The boundaries between the two P monomers are shown by yellow dashed lines. The locations of the histo-blood group antigens/oligsaccharides binding interfaces are labeled by yellow dashed circles.

For further understanding of the binding pocket, we compared the surface structures of known glycan binding interfaces of representative GI huNoVs. We noted that the P-loop and T-loop on the surface of DSV/VA115 P protein exhibit a higher extension and the distance between the two loops becomes closer (Fig. 6). Therefore, the glycan binding pocket becomes narrower but deeper, which hinders the binding to HBGAs. Despite the lack of a complex structure, previous studies have shown that GI.3 huNoVs Kashiwa_645, JKPG_883, and Osaka-bound HBGAs (30–32). Through sequence alignment analyses of the P domains among GI.3 huNoVs, we found the four critical amino acids related to glycan binding are highly conserved, but those around the glycan binding site are not (Fig. S5), suggesting that the morphological property of binding pocket could be changed.

The facts that DSV and VA115 do not bind HBGAs but can still infect humans indicates that the two GI.3 huNoVs must recognize molecules other than HBGAs as attachment factors. Our data demonstrate that DSV/VA115 indeed recognizes a group of glycans sharing a common terminal Gal, including Galα1-3Galβ1-4Glc and NA2 *N*-glycan. Significantly, the crystal structures of DSV P protein in complex with Galα1-3Galβ1-4Glc and NA2 *N*-Glycan, respectively, and the crystal structure of the VA115 P protein in complex with Galα1-3Galβ1-4Glc showed the interaction of DSV/VA115 with these glycans using the terminal Gal as the MaBS. These data verify the vital role of terminal Gal in the recognition by the two GI.3 huNoVs.

It has been known that glycan binding profiles of huNoVs affect their host susceptibility (41). We speculated that DSV and VA115 recognize a wide range of glycans containing terminal Gal, including Galα1-3Galβ1-4Glc, which exists at the end of isoglobotriaosylceramide (Galα1-3Galβ1-4Glcb1-1Cer). Isoglobotriaosylceramide, also known as iGb3 or Ganglioside Gv (A3), is a kind of glycosphingolipids present in the small intestine (42). Previous studies have demonstrated that iGb3 is involved in the interaction with enterohemorrhagic Escherichia coli and the Galα1-3Galβ1-4Glc is supposed as the basic element for recognition (43). At present, little is known regarding glycomics of the small intestinal mucosa, so distribution of glycans with terminal Gal in the human intestinal mucosal epithelium remains elusive, although it is speculated that there are several Gal-containing glycans that may also act as attachment factors. A recent study showing that GII.13 P protein interact with terminal Gal containing core-2 glycan (44) is another support to our conclusion.

Previous studies have shown that certain huNoVs have diverged from the traditional GI/GII lineages, probably due to the high mutation rate of the viral RNA genome. This divergent evolution might grant huNoVs the ability to escape from the herd immunity, as well as the possibility to extend their target population through recognition of new host factors. For example, the GII.13/21 strains have abandoned the conserved GII binding pocket and evolved a novel binding site, while still retaining the Lewis HBGA binding ability (45, 46). As for GI.3 huNoVs studied here, the location, composition, and binding mode remain conserved like other GI huNoVs, but the adjustment of surface loop regions has rendered the pocket unfit for HBGA recognition and binding. This phenomenon shows that although DSV and VA115 have abolished HBGA binding ability, host cell surface saccharides still pose a strong selection pressure on the evolution of huNoVs.

In summary, we have provided solid phenotypic and structural evidence to reasonably explain why these two GI.3 huNoVs lack HBGA binding capability but retain the ability to infect human hosts. The observed interactions between the two GI.3 huNoV P domains and Galα1-3Galβ1-4Glc and NA2 *N*-Glycan suggest that only the Gal-binding site is retained and GI.3 huNoVs may infect the human digestive tract through attaching to glycans with terminal Gal. The deeper and narrower binding pocket implies DSV/VA115 has stricter restrict on the attachment factor, which may be the reason why the two GI.3 P domains do not bind to any HBGAs experimentally.

## MATERIALS AND METHODS

### Production of huNoV P proteins.

The cDNA fragments encoding the P proteins of two clinical GI.3 huNoVs, the Desert Shield virus DSV395 (GenBank code: U04469.1, VP1 amino acids 227–544) and the VA98115/1998 (GenBank code: AY038598.1, VP1 amino acid 227–538), were chemically synthesized and cloned into expression vector pGEX-6P-1 at EcoRI and SalI*/*XhoI restriction sites. The P proteins were expressed in E. coli BL21(DE3) as described elsewhere (47–49). The resulting GST-P fusion proteins were purified using glutathione-Sepharose 4B (GE Healthcare Life Sciences) according to the manufacturer’s instructions, and the GST tag was removed with Prescission protease at 4°C overnight. The P proteins were further purified by anion ion exchange using Mono Q 5/50 GL (GE Healthcare Life Sciences) and gel-filtration chromatography using Superdex75 (GE Healthcare Life Sciences), in a buffer containing 20 mM HEPES pH 7.5 and 150 mM NaCl (Fig. S1A and B). Purified P proteins were concentrated to 8 mg/mL for crystallization. The P protein mutants of VA115 and DSV were constructed and purified using the same methods as for native P proteins.

### Glycan array screening.

The glycan array was performed at National Center for Functional Glycomics (NCFG; http://www.functionalglycomics.org) in America to identify potential glycan ligands that interact with VA115/DSV GST-P fusion proteins. The NCFG Mammalian Printed Array Library (version 5.1) containing 585 mammalian cell-associated glycans were tested. Each recombinant P-GST fusion protein was tested for binding to glycans using NCFG glycan microarray NHS slides purchased from Schott using previously published protocols (50–52). The GST-P protein samples were diluted at 5, 50, and 200 μg/mL and tested by anti-GST-488 antibody from Invitrogen (lot no. 1275883). The final results included the binding affinity of the proteins with individual glycans, measured by relative fluorescent units (RFUs) (52) and presented in histogram form as well as average RFU with standard deviation and percent coefficient of variance. The glycan array results are permanently stored in the public NCFG database (http://www.functionalglycomics.org/glycomics/publicdata/selectedScreens.jsp) with glycan array code: 3872 (VA115) and 3873 (DSV).

### Crystallization of P proteins and crystal soaking with oligosaccharides.

The native crystals of DSV and VA115 P proteins were grown by the hanging-drop vapor diffusion method, with the crystallization droplet containing 1 μL protein and 1 μL reservoir solution above a total of 200 μL reservoir solution. The reservoir solution for DSV P protein contained 15.5% (wt/vol) polyethylene glycol 3350 (PEG3350) and 0.2 M sodium tartrate dibasic dihydrate. The reservoir solution for VA115 P protein included 0.2 M sodium formate pH 6.4, 17% (wt/vol) PEG3350. Crystals were harvested within 7 days at 20°C. To obtain the complex of P protein and oligosaccharides, P protein crystals were soaked overnight in pre-equilibrated reservoir solution plus oligosaccharide, where final concentration of Galα1-3Galβ1-4Glc (G44766; Biosynth Carbosynth) was 20 mg/mL and NA2 *N*-Glycan (ON10165; Biosynth Carbosynth) was 10 mg/mL.

### X-ray diffraction data collection and processing.

Native and soaked protein crystals were briefly transferred to a cryoprotectant drop containing crystallization reservoir solution plus 15% (vol/vol) glycerol before flash frozen with liquid nitrogen. Native crystal and complexes with Galα1-3Galβ1-4Glc and NA2 *N*-Glycan for DSV/VA115 P proteins were collected at the rotating-anode X-ray source MicroMax-007/Satun 944 HG/Varimax HF (Institute of Biophysics, Chinese Academy of Sciences, Beijing, China) at a wavelength of 1.5418 Å. All data sets were collected under 100 K conditions and processed by HKL2000 (53). The statistics of the diffraction data are summarized in Table 1.

**TABLE 1:** Statistics for data collection and refinement of DSV and VA115 P protein structures

Parameter	DSV			VA115	
	Native	Galα1-3Galβ1-4Glc complex	NA2 *N*-glycan Complex	Native	Galα1-3Galβ1-4Glc complex
Data collection					
Resolution^*a*^	2.10 (2.14–2.10)	2.05 (2.09–2.05)	2.15 (2.19–2.15)	2.18 (2.24–2.20)	2.20 (2.24–2.20)
Completeness^*a*^	97.4 (76.2)	98.0 (81.7)	99.3 (90.9)	94.1 (67.9)	94.5 (73.4)
Mean I/sigma^*a*^	21.49 (4.15)	16.95 (1.71)	15.66 (1.91)	35.78 (5.92)	16.00 (4.81)
*R*_merge_ ^*a*^ ^,^ ^*b*^	0.086 (0.374)	0.121 (0.465)	0.173 (0.571)	0.076 (0.354)	0.081 (0.161)
No. of reflections	262,354	445,899	379,887	294,211	236380
No. of unique reflections	28,522	42,161	36,381	54,367	52,013
Refinement					
*R*_work_^*c*^/*R*_free_^*d*^	0.178/0.226	0.179/0.225	0.181/0.230	0.186/0.226	0.178/0.220
RMSD from ideal values					
Bond lengths (Å)	0.005	0.004	0.005	0.005	0.005
Bond angles (°)	1.314	1.258	1.300	1.325	1.321
avg B factors (Å^2^)					
Total	30.4	27.3	36.7	19.9	26.7
Protein	29.7	26.4	36.1	19.4	26.5
Ligand	–^*e*^	36.6	72.9	–	60.0
Solvent	36.7	34.0	39.0	24.1	28.3
Ramachandran plot (%)					
Favored	97.6	97.8	96.8	98.7	98.4
Allowed	2.4	2.2	3.2	1.3	1.6
Disallowed	0	0	0	0	0
PDB accession no.	7EQS	7EQT	7EQW	7ER0	7ER1

a Values in parentheses correspond to the shell of the highest resolution; RMSD, root mean square deviation; PDB, Protein Data Bank.

b *R*_merge_ = Σi|li−|/Σili, where i, li, and ili are the observed and mean intensity of related reflections with common indices *h*, *k*, and *l*.

c *R*_work_ = Σ‖*F*_obs_|−|*F*_cal_‖/Σ|*F*_obs_|, where *F*_obs_ and *F*_cal_ are observed and calculated structure factors, respectively.

d *R*_free_ = Σ*T*‖*F*_obs_|−|*F*_cal_‖/Σ*T*|*F*_obs_|, where *T* is a randomly selected test data set (~5%) of total reflections and was set aside before structure refinement.

e Dashes is an indicator of no B factors for non-existing ligands.

### Structure determination and refinement.

The Phaser program from CCP4 (54) was used to solve the crystal structures of DSV P protein and VA115 P protein by molecular replacement with the P protein of GI.7 Norovirus variant (Protein Data Bank [PDB] entry: 4P25) (20) as the searching model. The space group of DSV and VA115 P protein crystal was *P*2_1_2_1_2_1_ and *P*1, respectively. The amino acid sequence was then replaced respectively with that of DSV and VA115 P protein. Manual model building was done with COOT (55) and further refinement and adjustment were carried out with Phenix (56). Structures were validated with Procheck (57) and the structure analysis and presentation were generated by EdPDB (58) and PyMol (59). The complex structures of DSV P protein with Galα1-3Galβ1-4Glc and NA2 *N*-Glycan, as well as VA115 P protein with Galα1-3Galβ1-4Glc, were processed in the same way, with respective native structure as initial model.

### Mutation validation and microscale thermophoresis binding analyses.

In order to verify amino acids that contribute to interaction with oligosaccharides, DSV/VA115 native and mutant P proteins were prepared to test the interaction with Galα1-3Galβ1-4Glc-HAS (NGP2330; Detra) by mutation validation and MST analyses (60). Dilution buffer was 20 mM HEPES pH 7.5, 150 mM NaCl, and 0.05% (vol/vol) Tween 20. The Galα1-3Galβ1-4Glc-HSA was labeled following the protocol of the Protein labeling kit RED-NHS (L011; Nanotemper). For the DSV/VA115 native and mutant P proteins, 16 2-fold serial dilutions beginning at different concentrations (at least 20 μM) were prepared. The labeled Galα1-3Galβ1-4Glc-HSA (final concentration at 20 nM) were mixed with various concentrations of candidate proteins at equal volume. After 5 min of incubation at room temperature, the mixture was aspirated into glass capillaries for measurement. The MST experiment was performed using Monolith NT.115 instrument (NanoTemper Technologies), and data were collected under 40% infrared laser power and 40% excitation power. Each MST assay included three or four groups of parallel experiments, and the average value was taken to fit the combination curve. The data were analyzed by MO.Affinity Analysis v2.3 and the *K*_D_ was calculated.

### Data availability.

The coordinates and structure factors of the DSV and VA115 native P proteins and the complexes with Gala1-3Galß1-4Glc and/or NA2 *N*-glycan have been deposited in the PDB under accession numbers 7EQS, 7EQT, 7EQW, 7ER0, and 7ER1 (Table 1). The glycan array results are permanently stored in the public NCFG database (http://www.functionalglycomics.org/glycomics/publicdata/selectedScreens.jsp) with glycan array codes 3872 (VA115) and 3873 (DSV).
