# Supplementary material for: Structural Insight into Terminal Galactose Recognition by Two Non-HBGA Binding GI.3 Noroviruses
Source: J Virol. 2022 Jun 6;96(13):e00420-22. doi: 10.1128/jvi.00420-22 (PMC9278146; doi:10.1128/jvi.00420-22)
Supplement: Supplemental file 1 — Fig. S1 to S4. Download jvi.00420-22-s0001.pdf, PDF file, 1.7 MB [file jvi.00420-22-s0001.pdf]

Supplementary information

Structural insight into terminal galactose recognition by two non-HBGA binding GI.3 noroviruses

C. Wang et al.

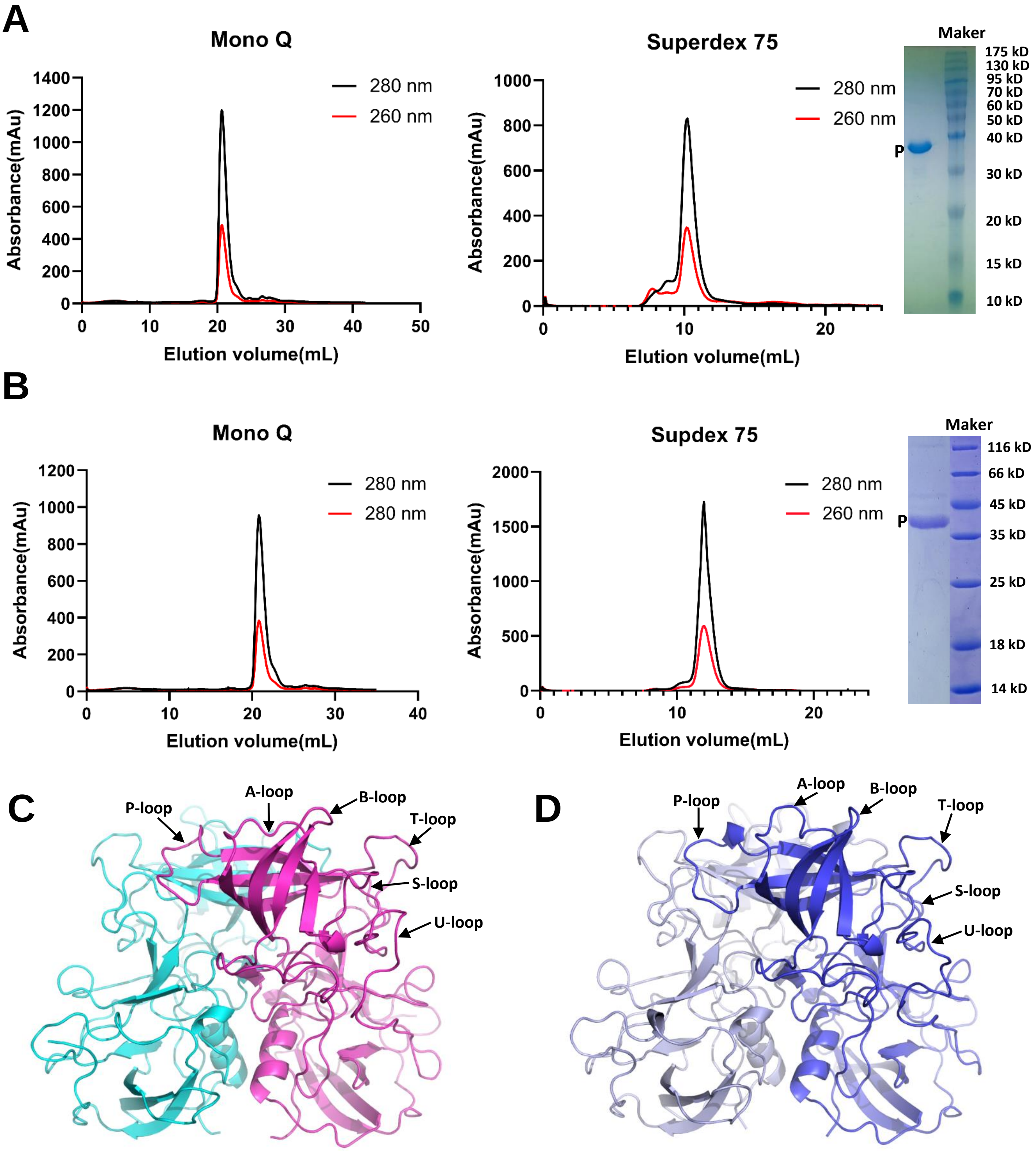

S1 Fig. Protein purification and native structures of native GI.3 P proteins.

(A and B) Productions of the DSV (A) and VA115 (B) P proteins via the *E. coli* expression system. Shown elution profile of DSV (A) and VA115(B) P protein on the Mono Q 5/50 GL anion ion exchange and Superdex75 10/300 GL size-exclusion column (repeated ≥ 5 times).The purified P proteins (~35 kDa) are analyzed by SDS-PAGE. **(C and D)** Cartoon representation of the native DSV P dimer (C) and native VA115 P dimer (D) at the side view.

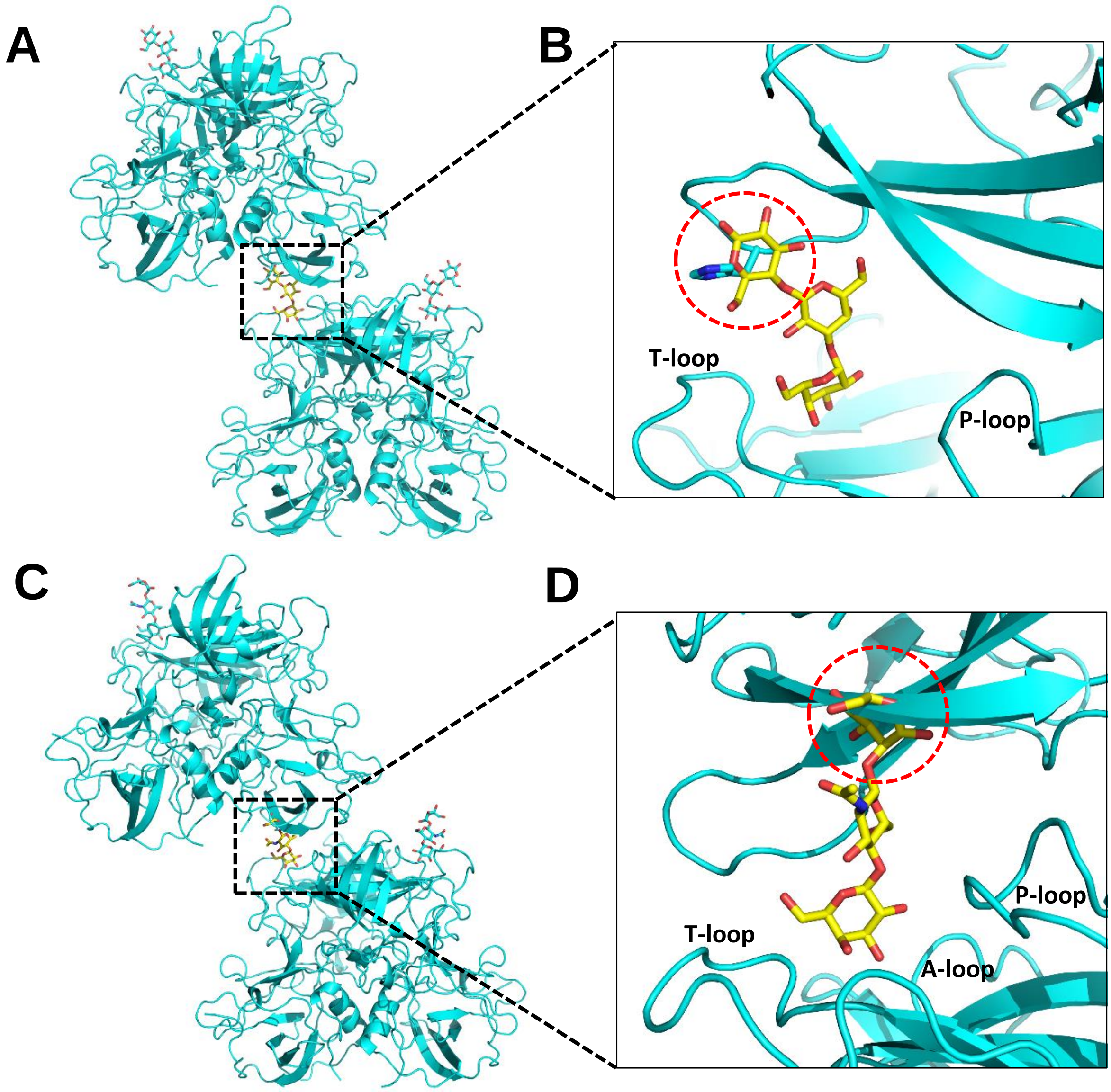

S2 Fig. Steric hindrance for glycans at P protein crystal packing interface. Displayed the asymmetric DSV P dimer, and simulated Galα1-3Galβ1-4Glc (A and B) and NA2 N-Glycan (C and D) into another oligosaccharide binding pocket. Found that both of them have chemical steric hindrances, which were indicated by red dotted circles.

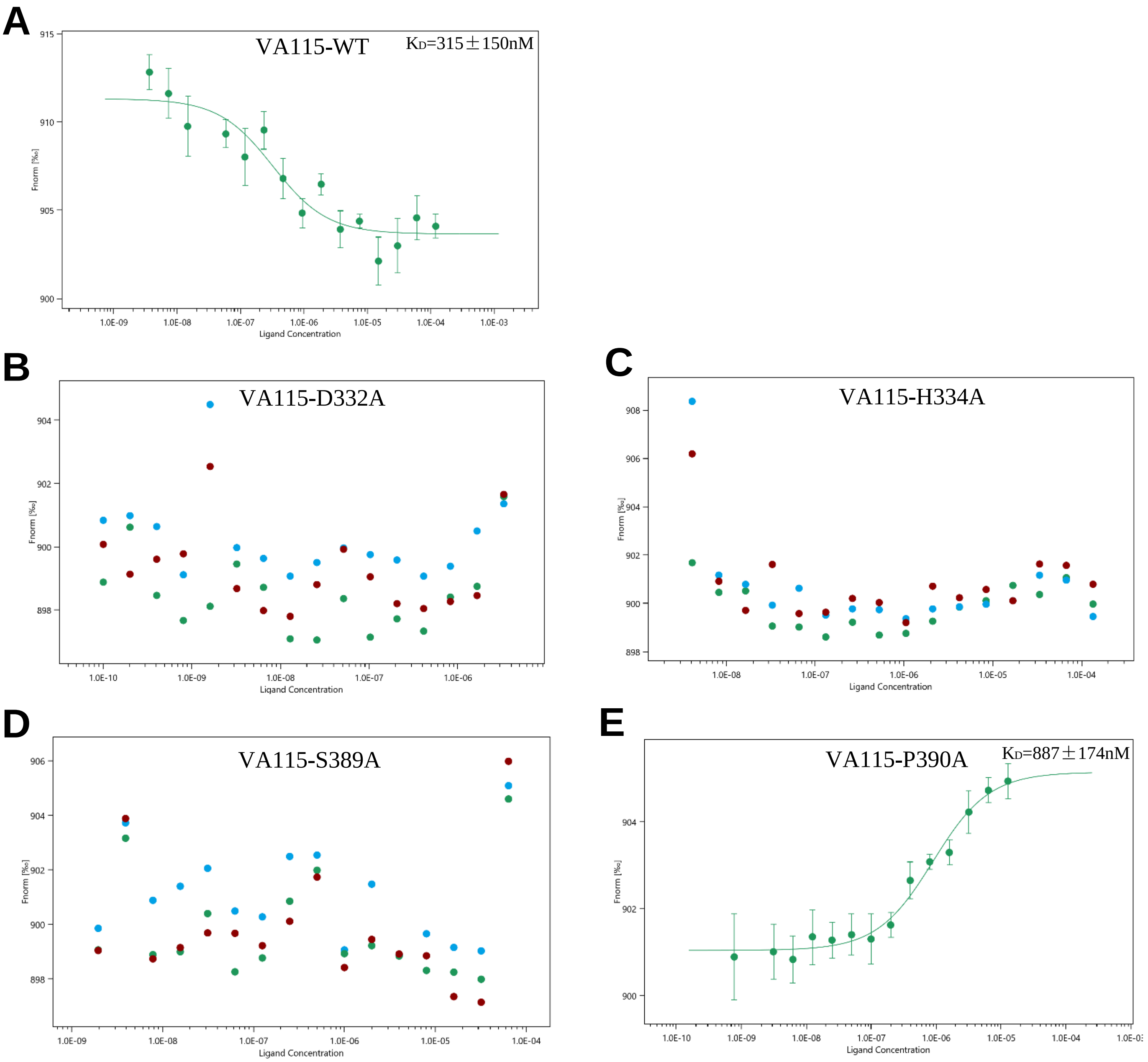
A
VA115-WT
KD=315±150nM
Fnorm [‰]
Ligand Concentration
B
VA115-D332A
Fnorm [‰]
Ligand Concentration
C
VA115-H334A
Fnorm [‰]
Ligand Concentration
D
VA115-S389A
Fnorm [‰]
Ligand Concentration
E
VA115-P390A
KD=887±174nM
Fnorm [‰]
Ligand Concentration

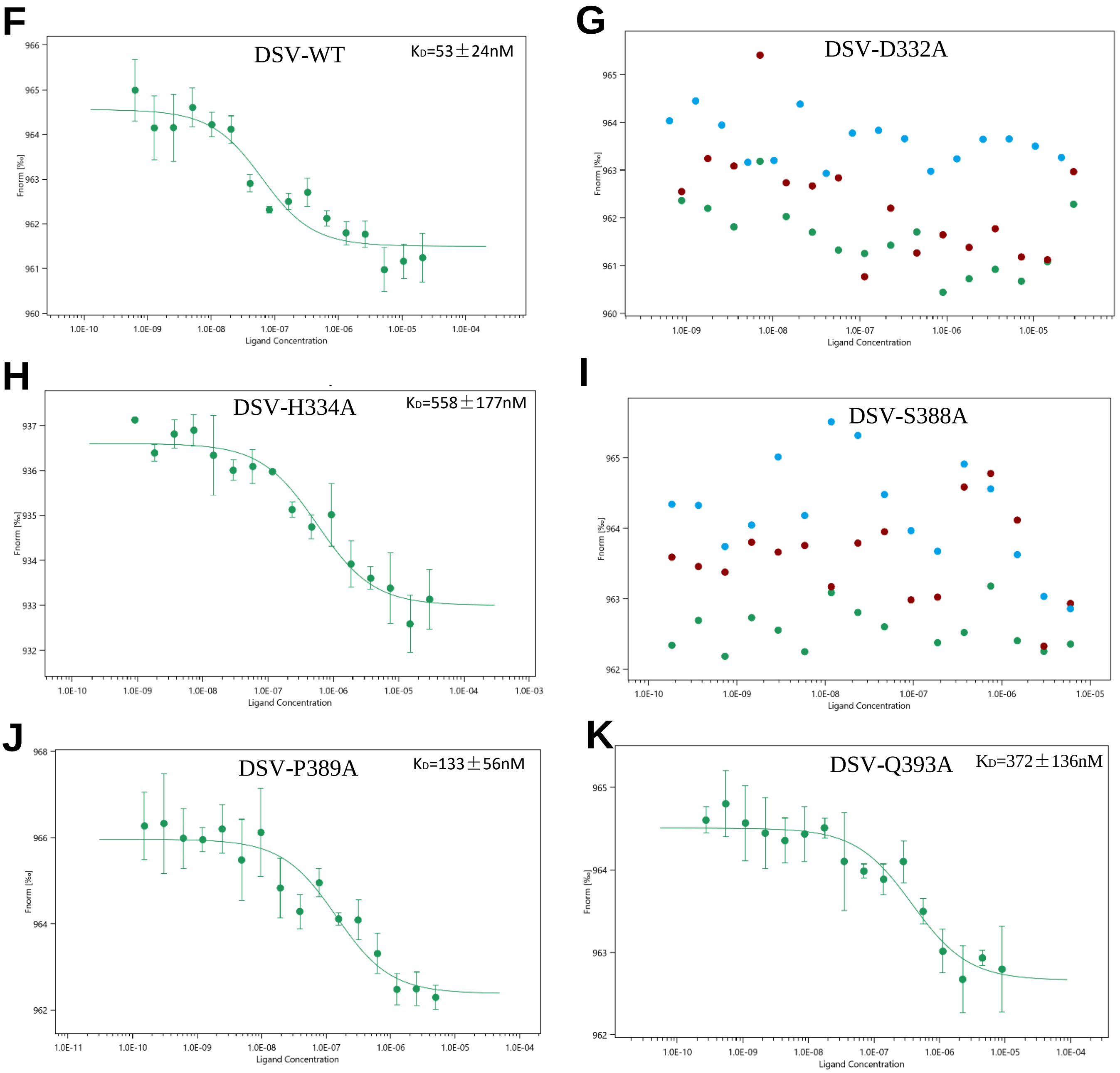

S3 Fig. Galα1-3Galβ1-4Glc binding outcomes of wild type and various mutant P proteins with single amino acid mutations in the Galα1-3Galβ1-4Glc binding interface of DSV and VA115.

(A–E) Galα1-3Galβ1-4Glc binding outcomes of wild type (A) and various mutant P proteins with single amino acid mutations in the Galα1-3Galβ1-4Glc binding interface of VA115. **(F–K)** Galα1-3Galβ1-4Glc binding outcomes of wild type (F) and various mutant P proteins with single amino acid mutations in the Galα1-3Galβ1-4Glc binding interface of DSV. X axes indicate the ligand concentrations (P proteins), while the Y-axes indicate the fluorescence (‰). Reliable binding signals were detected only between VA115-WT, VA115-P390A, DSV-WT, DSV-H334A, DSV-P389A, DSV-Q393A and Galα1-3Galβ1-4Glc, respectively. The equilibrium dissociation constants (KD) are315±150 nM, 887 ± 174 nM, 53 ± 24 nM, 558 ± 177 nM, 133 ± 56 nM and 372 ± 136 nM, respectively.

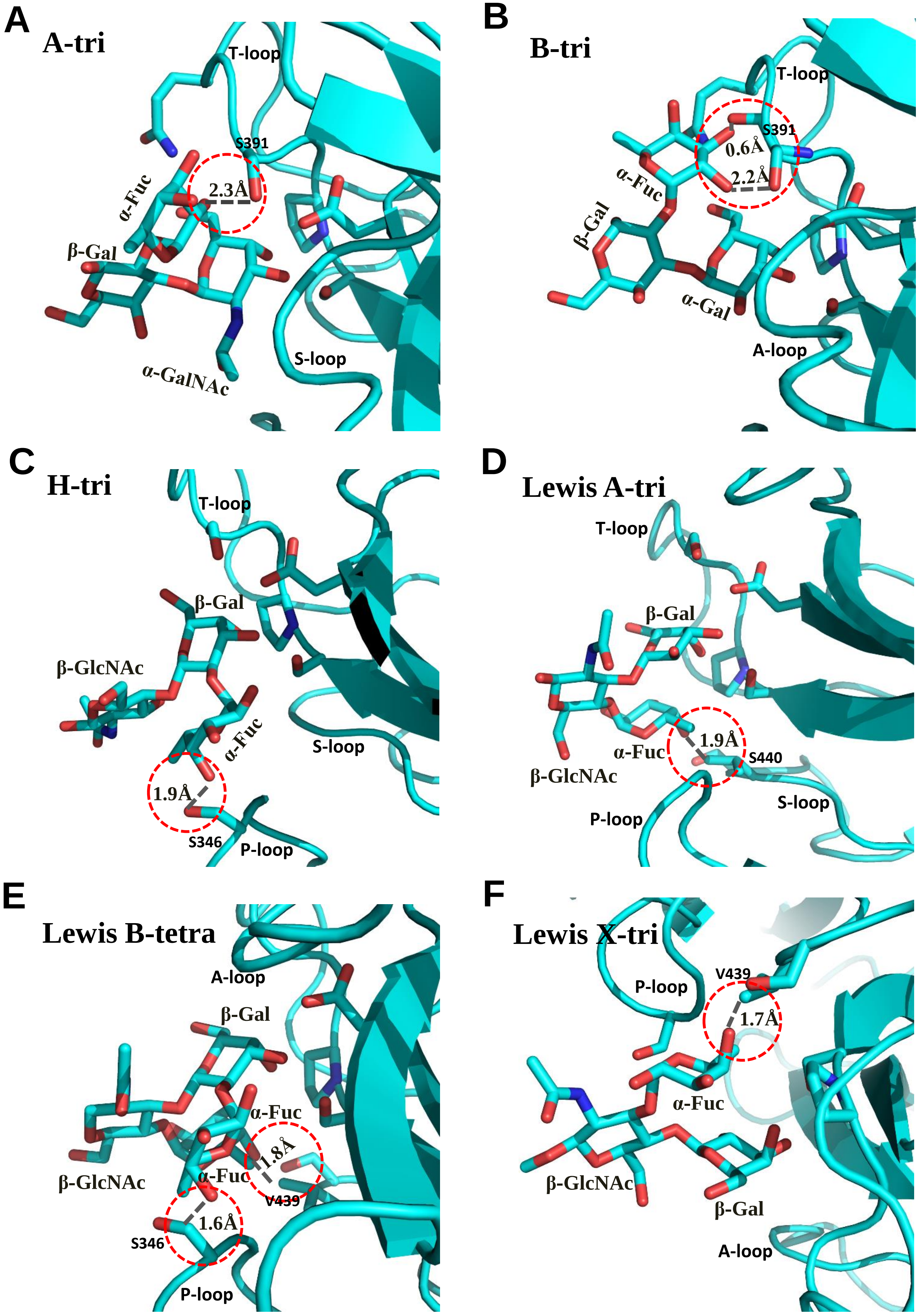
A
A-tri
T-loop
S391
2.3Å
α-Fuc
β-Gal
α-GalNAc
S-loop
B
B-tri
T-loop
S391
0.6Å
2.2Å
α-Fuc
β-Gal
α-Gal
A-loop
C
H-tri
T-loop
β-Gal
β-GlcNAc
α-Fuc
S-loop
1.9Å
S346
P-loop
D
Lewis A-tri
T-loop
β-Gal
1.9Å
α-Fuc
S440
β-GlcNAc
S-loop
P-loop
E
Lewis B-tetra
A-loop
β-Gal
α-Fuc
β-GlcNAc
1.8Å
α-Fuc
V439
1.6Å
S346
P-loop
F
Lewis X-tri
P-loop
V439
1.7Å
α-Fuc
β-GlcNAc
β-Gal
A-loop

G

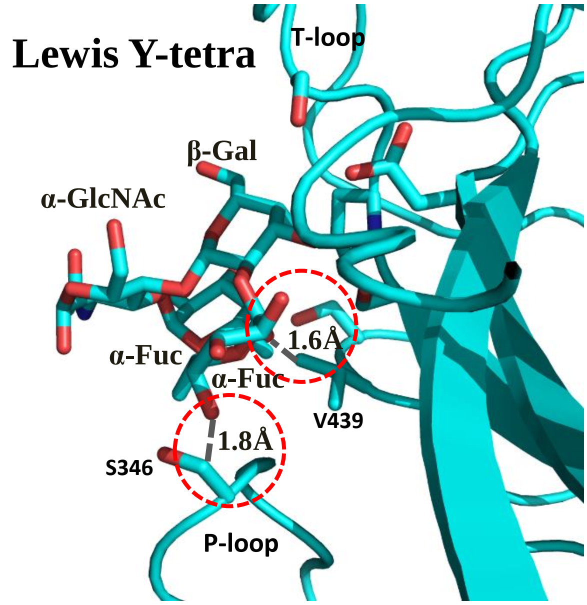

S4 Fig. Cartoon views of DSV P-NA2 N-Glycan complex structure and oligosaccharides (representing types A, B, H, Le^{a}, Le^{b}, Le^{x} and Le^{y}, respectively) superposition. Structural superposition is performed by superposition of galactoses.

(A-G) Show the chemical steric hindrances between DSV P dimer and type A trisaccharide (A), type B trisaccharide (B), type H trisaccharide (C), Lewis A trisaccharide (D), Lewis B tetrasaccharide (E), Lewis X tetrasaccharide (F) and Lewis Y trisaccharide (G), respectively. These oligosaccharides and residues involved in the interactions are shown in stick representation. The steric hindrancesis represented by grey dashed lines and labeled by red dashed circles. The structure of type A trisaccharide , type B trisaccharide, type H trisaccharide, Lewis A trisaccharide, Lewis B tetrasaccharide, Lewis X trisaccharide and Lewis Y tetrasaccharide are came from PDB:2ZL7, PDB:3Q38, PDB:4P1V, PDB:3ASR, PDB:3ASS, PDB:1UZ8 and PDB:4RDL, respectively.
